# Consensus on the need for a hierarchical list of patient-reported pain outcomes for meta-analyses of knee osteoarthritis trials

**DOI:** 10.1186/1745-6215-16-S1-P36

**Published:** 2015-05-29

**Authors:** Louise Klokker, Lara J Maxwell, Peter Juni, David Tovey, Paula R Williamson, Maarten Boers, Niti Goel, Rachelle Buchbinder, Lyn March, Caroline B Terwee, Jasvinder A Singh, Peter Tugwell, Robin Christensen

**Affiliations:** 1 Musculoskeletal Statistics Unit, The Parker Institute, Dept. Rheum, Copenhagen University Hospital, Bispebjerg and Frederiksberg, Denmark; 2 Institute of Population Health, University of Ottawa, Ottawa, Ontario, Canada; 3 Institute of Primary Health Care (BIHAM) and Clinical Trials Unit Bern, University of Bern, Switzerland; 4 Cochrane Collaboration, London, United Kingdom; 5 MRC North West Hub for Trials Methodology Research, Department of Biostatistics, University of Liverpool, Liverpool, United Kingdom; 6 Departments of Epidemiology and Biostatistics, and Rheumatology, VU University Medical Center, Amsterdam, the Netherlands; 7 Quintiles Inc; Division of Rheumatology, Department of Medicine, Duke University School of Medicine; Patient research partner; Durham, North Carolina, USA; 8 Department of Epidemiology and Preventive Medicine, School of Public Health and Preventive Medicine, Monash University, Melbourne and Monash Department of Clinical Epidemiology, Cabrini Health, Melbourne, Australia; 9 Northern Clinical School, Institute of Bone and Joint Research, University of Sydney, Department of Rheumatology, Australia; 10 Department of Epidemiology and Biostatistics and the EMGO Institute for Health and Care research, VU University Medical Center Amsterdam, the Netherlands; 11 Birmingham Veterans Affairs Medical Center and University of Alabama at Birmingham, Birmingham, Alabama, USA; 12 The Department of Medicine, University of Ottawa, Ontario, Canada

## 

The selection of appropriate outcomes is crucial when designing, and subsequently interpreting clinical trials, in order to directly compare the effects of different interventions in ways that minimize bias. The same is likely to apply for systematic reviews and meta-analyses. Although protocol registration for systematic reviews is still not mandatory, reviewers should be strongly encouraged to register the protocol, in order to identify - a priori - the proposed methodological approach, including all outcomes of interest. This will help to minimize the likelihood of biased post hoc decisions in review methods, such as selective outcome reporting.

A group of international experts convened to address issues regarding the need to develop hierarchical lists of outcome measurement instruments for a particular outcome for meta-analyses. Meta-analysis of knee osteoarthritis (OA) trials, and the assessment of pain as an outcome, was used as an exemplar to assess how ‘Outcome Measures in Rheumatology’ (OMERACT) and other international initiatives might contribute in this area. The meeting began with formal presentations of background topics, empirical evidence from the literature, and a brief introduction to two existing hierarchical lists of pain outcome measures recommended for meta-analyses of knee OA trials.

After discussions most participants agreed that there is a need to develop a methodology for generation of hierarchical lists of outcome instruments for use to guide meta-analyses. Tools that could be used to steer development of such a prioritized list are the COSMIN checklist and the OMERACT filter 2.0. For future research we suggest that among outcome instruments frequently reported in trials for the same domain, those with the best measurement properties (e.g., validity and reliability) would achieve high, if not the highest rankings for use on a hierarchical list.

